# Rapid phenotypic differentiation in the iconic Japanese knotweed *s.l.* invading novel habitats

**DOI:** 10.1038/s41598-024-64109-1

**Published:** 2024-06-25

**Authors:** Wei Yuan, Massimo Pigliucci, Christina L. Richards

**Affiliations:** 1 Department of Molecular Biology, Max Planck Institute for Biology, Tübingen, Germany; 2 Department of Philosophy, City College of New York, New York, NY USA; 3 Plant Evolutionary Ecology Group, University of Tübingen, Tübingen, Germany; 4 Department of Integrative Biology, University of South Florida, Tampa, FL USA

**Keywords:** Adaptive evolution, Hybridization, Natural selection, Phenotypic differentiation, Conservation biology, Ecological genetics

## Abstract

Understanding the mechanisms that underlie plant invasions is critical for management and conservation of biodiversity. At the same time, invasive species also provide a unique opportunity to study rapid adaptation to complex environmental conditions. Using four replicate reciprocal transplant experiments across three habitats, we described patterns of phenotypic response and assessed the degree of local adaptation in knotweed populations. We found plants from beach habitats were generally smaller than plants from marsh and roadside habitats when grown in their home habitat. In the marsh habitat, marsh plants were generally larger than beach plants, but not different from roadside plants. There were no differences among plants grown in the roadside habitat. We found mixed evidence for local adaptation: plants from the marsh habitat had greater biomass in their “home” sites, while plants from beaches and roadsides had greater survival in their “home” sites compared to other plants. In sum, we found phenotypic differentiation and some support for the hypothesis of rapid local adaptation of plants from beach, marsh and roadside habitats. Identifying whether these patterns of differentiation result from genetic or heritable non-genetic mechanisms will require further work.

## Introduction

Conservation efforts have a long history of relying on genetic information to strategically protect biodiversity^1,2^. This has often taken the form of documenting the potential effects of inbreeding and genetic drift in small populations, or the importance of diversity as an indicator of resilience in the face of environmental change. At the same time, the Convention on Biological Diversity^3^ recognizes that invasive species are “main direct drivers of biodiversity loss across the globe”. Despite decades of work to understand invasion, the ecology of most invasive species remains poorly understood^4^. As with rare and endangered species, genetics approaches promise to provide insight into the mechanisms that allow for invasive species to spread. Application of such information to the management of invasive species will ultimately contribute to the preservation of biodiversity. Invasive species also provide a unique opportunity to understand the process of adaptation despite the likelihood of reduced genetic variation following a population bottleneck^5,6^.

The classic population genetic assumption is that dramatically reduced genetic variation will severely constrain the evolutionary potential of a given population or species^7,8^. The chances of a few individuals or only one genotype landing in a novel location and surviving are expected to be minimal^9,10^. Some invasive species benefit from alternative sources of increased genetic variation through multiple introductions^11–13^ or hybridization^14–18^. However, many invasive species appear to do well even with low levels of sequence-based variation^19–22^. This could be through chance establishment of “general purpose genotypes”^23–25^, high performance genotypes, or rapid evolution of niche breadth^26,27^. Van Kleunen et al*.*^4^ argued that adaptive evolutionary processes are at least as common in invasive species as native species, which is surprising given the short amount of time they have to evolve. In fact, a meta-analysis of 134 plant species in 52 plant families showed that invasive plant species demonstrated patterns of local adaptation just as frequently, and at least as strongly as native plant species^28^.

Given the limited genetic diversity of many invasive populations, phenotypic plasticity has often been suggested as a potentially important mechanism in the invasion process^23,29–31^. Many studies have found support for the importance of plasticity^21,26,32–34^ (but see Ref.^35^). Moreover, recent work has explored how epigenetic mechanisms may contribute to the persistence of such plastic responses across generations^36–39^ and may be especially relevant in response to stressful or novel environments^40^. The chance sampling of individuals, combined with non-genetic sources of phenotypic variation, can lead to divergence in phenotypes of invasive populations even in the absence of abundant inter-individual genetic variation^41–43^. Considering the plurality of potential mechanisms of rapid evolution in novel conditions, studies that examine phenotypic responses of clonal replicates in natural settings will enhance our understanding of the processes of adaptation.

Invasive populations of the *Reynoutria* species complex (referred to as Japanese knotweed sensu lato or *s.l.*) are known to occupy a wide range of habitats in Europe^15,44–46^, and the United States^47–50^. On Long Island, NY, in addition to roadside habitats and along railways, Japanese knotweed is often growing on beaches and next to or on the terrestrial border of marshes that have been invaded by *Phragmites australis*^48,51,52^. These populations provide a natural laboratory to explore rapid adaptation to very different habitat types. The physiological challenges of living in beach and salt marsh habitats might require a dramatic phenotypic shift. For example, plants that are able to tolerate highly saline environments are characterized by traits that specifically ameliorate the toxic and osmotic effects of substrate salinity to allow for growth under these conditions^53–56^.

Although the traits that allow for response to challenging conditions have been studied in a variety of taxa, response to such selective pressures may be constrained by a lack of heritable variation in these traits. In a previous study, we used cytology and AFLP markers to show that populations around Long Island, NY have extremely low genetic diversity: some are made up of a single *R. japonica* genotype reported across the US and Europe^19,46,50,57^, while the majority consist of a few *R.* × *bohemica* hybrid haplotypes^48,51,52^. Plants from both roadside and marsh habitats had highly plastic responses to treatment with salt, and despite the lack of diversity at the genotype level, we found significant differences in most traits and trait plasticities within and among sites^51^.

While controlled experiments allow us to examine response to specific environmental factors, reciprocal transplantation in natural field settings is the most robust approach to identify local adaptation to complex real environments^58^. Plants are considered to be locally adapted if they perform better in their “local site” or “home site” than do plants from other “foreign” sites. A robust quantitative genetics design in a transplant study also allows for investigating which traits are under selection^59–63^, and characterizing the patterns of divergence of traits across different habitats^64–67^.

If invasive Japanese knotweed plants have heritable variation for traits that are associated with increased fitness in these habitats, selection should act on these traits and lead to locally adapted populations. If these populations are adapted, we expect that they will outperform plants from other habitats, through greater biomass or greater survival, when grown in their local habitat^58^. In this study, we established reciprocal transplants among beach, marsh and roadside habitats, planting replicates of each individual back into its home site and into the other two habitat types to answer the following questions: (1) How do knotweed traits vary in response to the different habitat types? and (2) Does knotweed demonstrate patterns of adaptation to the different habitat types?

## Methods

### Reynoutria species complex

The taxonomy of Japanese knotweeds has been complicated^68–70^. Briefly, two species *Reynoutria japonica* and *R. sachalinensis* were originally from Japan and were introduced to Europe in the mid 1800’s and the United States by the end of the nineteenth century^71^. Although *R. sachalinensis* (2n = 44, 66, 88 and 132)^72,73^ is distinct morphologically from *R. japonica* (2n = 44 or 88), the two species are not differentiated in chloroplast DNA (cpDNA)^74^. Hybridization between them appears to be rare in Japan, where they are not usually sympatric (but see Ref.^72^ for evidence of introgression). However, the hybrid *R.* × *bohemica* is common in the invasive range in Europe^16,75–77^ and the US^52,78,79^. In the US, studies in New England suggested that spread of all three taxa takes place through both vegetative and sexual reproduction^49,78,79^. However, other studies in the U.S. on *R. japonica* report only the same single female genotype that has also been found throughout Europe^50,52,77^.

### Collection sites and experimental gardens

**Figure 1:**
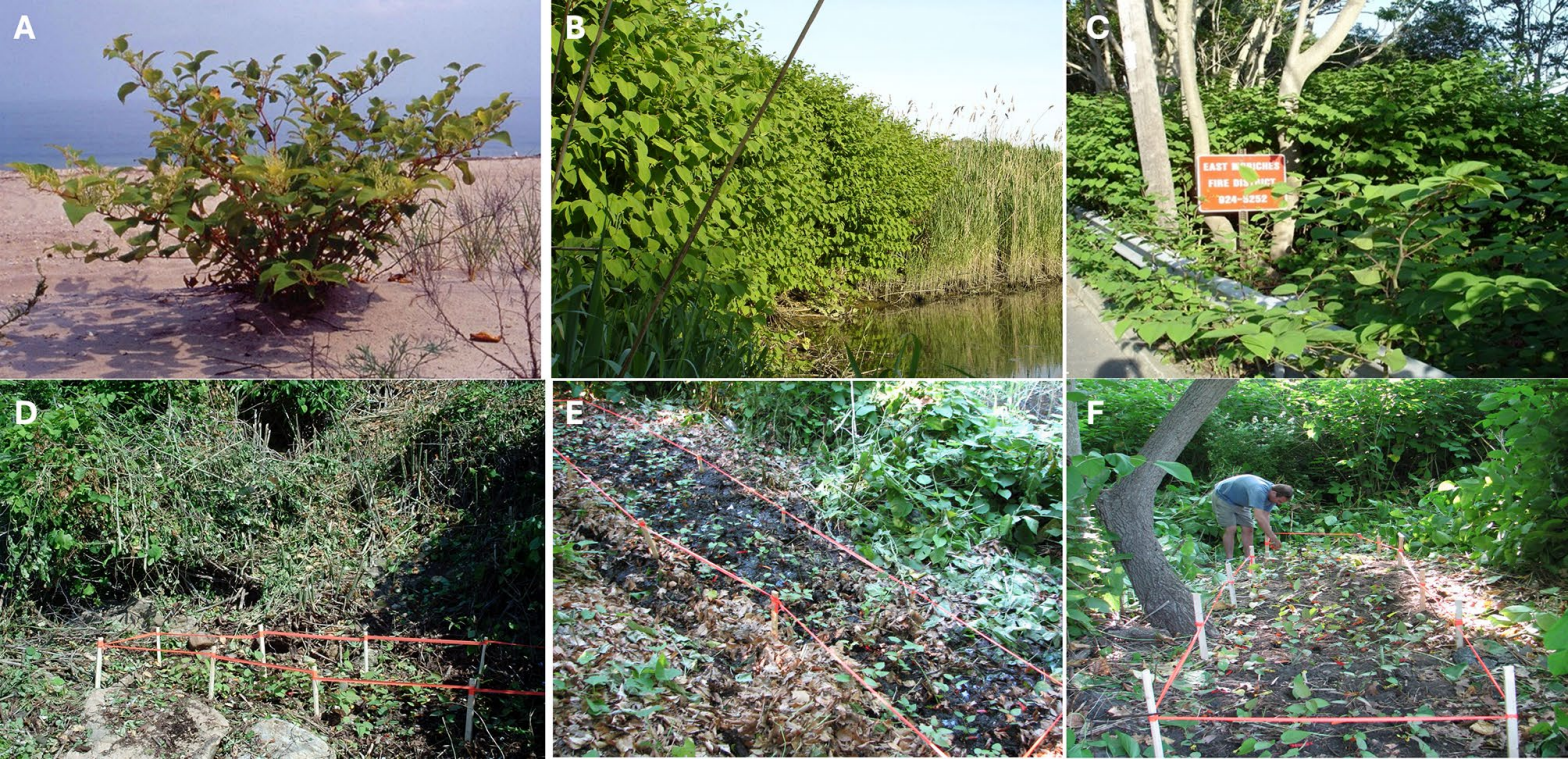
The three habitat types on Long Island that support introduced Japanese knotweed: (**A**). Beach habitats represented here by the Port Jefferson Beach (PJB) site. (**B**). Marsh habitats represented here by the Center Moriches Marsh (CMM) site. (**C**). Roadside habitats represented here by the Center Moriches Roadside (CMR) site. We cleared standing knotweed to set up transplant gardens in each habitat: (**D**). Beach transplant gardens represented here by the Horton’s Point (HP) site. (**E**). Marsh habitats represented here by the Crystal Brook Hollow (CBH) site. (**F**). Roadside transplant gardens represented here by the Riverhead Chauncey (RHC) site.

In mid-May 2005, we collected Japanese knotweed *s.l.* rhizomes from beach, marsh and roadside sites (e.g., Fig. 1A–C) across Suffolk County, Long Island, New York (Table 1) with permission from the Long Island National Wildlife Refuge, the Village of Port Jefferson, the Town of Southold and several private property owners. Voucher specimens of the species, including from Suffolk County, have been previously deposited at the Steere Herbarium of the New York Botanical Garden and the Brooklyn Botanic Garden Herbarium. Dr. Ramona Walls performed the initial formal identification of the plant material from these populations in previous work^48,51^. The experiment complied with relevant institutional, national, and international guidelines and legislation.

**Table 1:** Locations of origin sites for the three habitat types within each transplant garden group for the 12 sites (used as origins s for plant material and as locations of transplant gardens).

Transplant garden group	Origin site (habitat)	City	Latitude	Longitude	No. of rhizomes (reps/rhizome)	Total no. ramets	Survivors
1	MSH (Beach1)	Port Jefferson	40 57.7	73 02.6	6 (9–21)	81	35 (3, 25, 7)
1	CBH (Marsh 1)	Port Jefferson	40 57.2	73 02.7	5 (6–21)	117	32 (2, 24, 6)
1	ST (Roadside 1)	Smithtown	40 51.5	73 12.6	7 (6–15)	117	43 (0, 27, 16)
Total for transplant 1					18	315	110 (35%)
2	PJB (Beach 2)	Port Jefferson	40 57.9	73 03.2	7 (9–21)	93	19 (0, 5, 14)
2	CMM (Marsh 2)	Center Moriches	40 48.0	72 46.4	8 (9–24)	75	8 (0, 0, 8)
2	CMR (Roadside 2)	Center Moriches	40 48.0	72 46.4	8 (6–21)	72	8 (0, 0, 8)
Total for transplant 2					23	240	35 (15%)
3	RPB (Beach 3)	Rocky Point	40 58.0	72 57.3	6 (6–21)	147	37 (2, 12, 23)
3	WH (Marsh 3)	Brookhaven	40 46.2	72 53.9	8 (6–21)	117	8 (0, 4, 4)
3	HL (Roadside 3)	Rocky Point	40 57.8	72 57.3	8 (6–21)	111	35 (2, 17, 16)
Total for transplant 3					22	375	80 (21%)
4	HP (Beach 4)	Southold	41 05.2	72 26.7	8 (12–24)	120	39 (11, 6, 22)
4	RHBH (Marsh 4)	Riverhead	40 54.2	72 37.1	8 (6–19)	132	76 (22, 29, 32)
4	RHC (Roadside 4)	Riverhead	40 54.5	72 37.5	8 (9–21)	105	50 (9, 16, 25)
Total for transplant 4					24	357	165 (46%)

The number of rhizomes from each site (with range of replicates) and total number of replicates per site are provided, as well as the total number of rhizomes and replicates within the four reciprocal transplants. The total number of surviving plants from each origin site (and number of survivors in beach, marsh and roadside transplant gardens) is also indicated.

**Figure 2:**
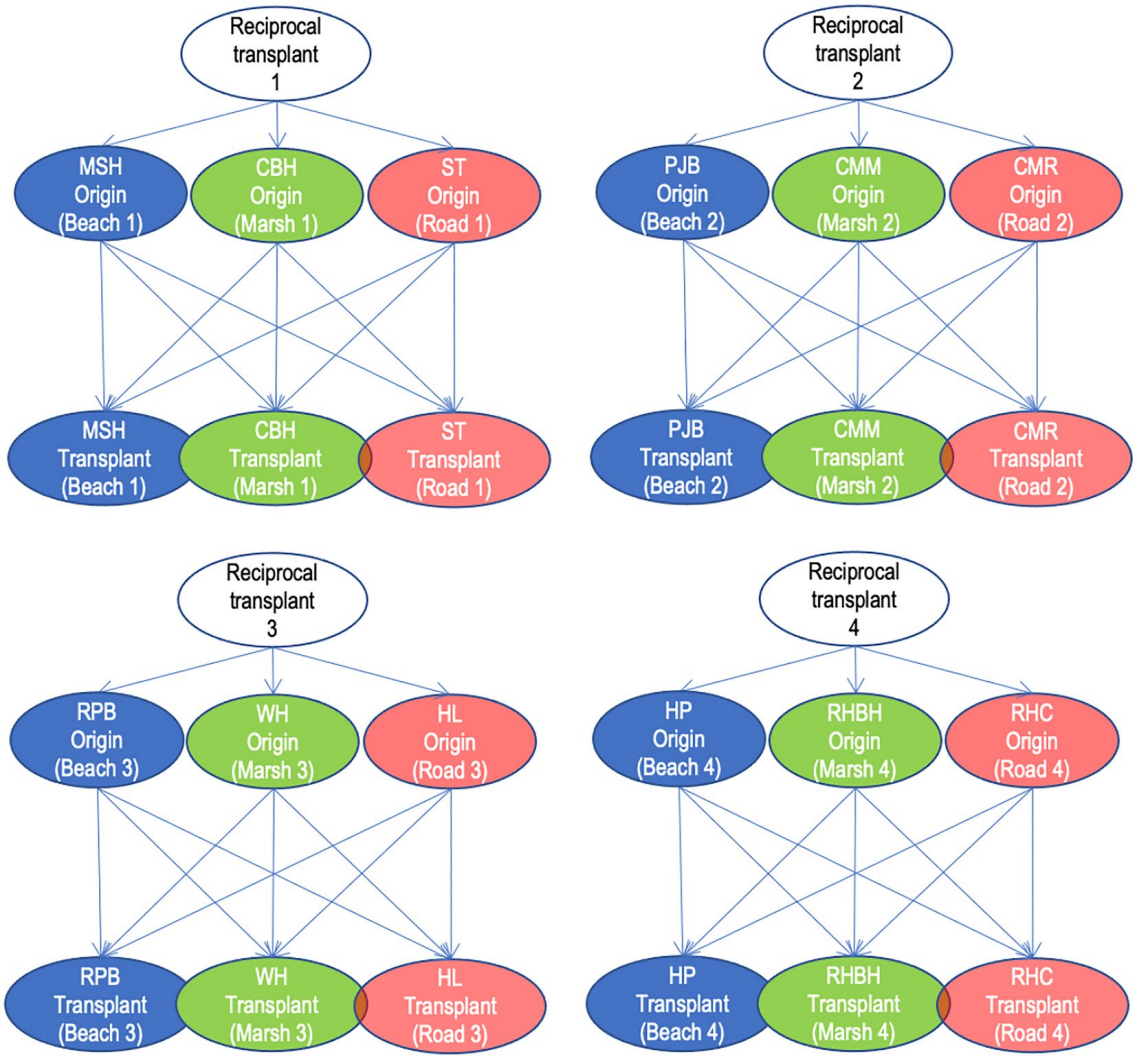
Design of the four reciprocal transplant experiments between beach, marsh, and roadside habitat sites around Long Island, NY. We used four sites of each habitat type for 12 total sites as origins for plant material and as locations of transplant gardens (see also Ref.^80^). By creating four reciprocal transplant experiments, we maximized our ability to replicate cuttings of each rhizome in one site of each of the habitats and test for superior performance in the original “local” site.

Reciprocal transplants are the gold standard to test for local adaptation, but they are rarely replicated across habitats. We identified four sites each of beach, marsh and roadside habitats (12 total sites) to establish four replicate reciprocal transplant experiments (see Figs. 1D–F, 2).

Due to the natural topography of Long Island, the beach sites are all located on the northern shore of Long Island, while the salt marsh sites and roadside sites are more evenly distributed around Suffolk County. We haphazardly chose sites to represent the three habitats. The beach sites are separated by 1–65 km, the marsh sites are separated by 14–40 km, and the roadside sites are separated by 20–32 km. At each site, we collected approximately one meter of rhizome from seven (at Beach 1) or eight individuals (at the other 11 sites) that were approximately 10 m apart, to maximize the chances of sampling different genotypes and to represent plant distribution at each site. However, our previous studies showed that almost all individuals collected within a site had the same AFLP haplotype and most likely were originally connected at some point as one individual.

We brought the rhizomes to the Stony Brook University greenhouse and cut them into pieces of 4–8 g fresh-weight (12 sites × 7 or 8 rhizomes per site for a total of 95 rhizomes × 18–25 replicates = 2225 rhizome pieces). Our analyses showed that this variation in initial rhizome weight did not significantly predict variation for any trait (see also^51^). We planted the rhizome pieces in individual wells in 24-well flats with Pro-mix potting medium (Pro-mix Bx, Quakertown, Pennsylvania, USA), and approximately one teaspoon of slow release fertilizer (15–9–12 Osmocote Plus 8–9 months, Marysville, Ohio). The flats were placed in a temperature-controlled greenhouse under conditions approximating mid-summer in Suffolk County, Long Island and watered as needed to keep the soil moist. Day temperature was maintained at 30 °C and night temperature at 25 °C. We grew the plants in the greenhouse for approximately six weeks to allow for shoots to emerge from the rhizomes and grow to a height of approximately 10–15 cm.

We organized the sites into four reciprocal transplant groups, each with one beach, one marsh and one roadside location, which were not necessarily in close proximity because of the natural distribution of the plant populations. This design allowed us to maximize our ability to replicate cuttings of each rhizome in one site of each of the habitat types and test for superior performance in the original “local” site. Within each group, we haphazardly assigned equal numbers of replicates of each rhizome to be transplanted into its home site and into one site of each of the other two habitat types. At each transplant site, we divided the garden into five contiguous blocks to be able to account statistically for within site variation (modeled as blocks in the linear models). Across the five blocks, we randomly assigned the position of the replicates of each rhizome (2–8 replicates per rhizome). Each of the 12 transplant gardens contained approximately 80–125 individuals for a total of 1287 plants (4 replicate studies × 3 transplant garden habitats × 3 origin habitats × 5–8 rhizomes × 2–8 replicates; Table 1). We prepared the transplant gardens by removing only above ground vegetation (typically other knotweed plants) to a height of less than 2 cm with a machete. The cleared area included a border of 15–30 cm outside of the transplant blocks.

Between June 16–21, we transplanted all plants into the field. In order to minimize transplant shock, we covered the beach garden plants on transplant to protect them from conditions that could be potentially too warm for the first few weeks. Otherwise, plants were minimally maintained. We marked the gardens with stakes and flagging, and we were easily able to keep track of the plants which we left in the field from the summer of 2005 through the fall of 2006. We evaluated initial survival in September of 2005 and found that about half (ranging from 46 to 86%) of the plants in each transplant garden had successfully established in the first year. We measured the final height and final number of leaves on all plants and harvested above ground and below ground for all plants between 11 and 18 September 2006. We were able to separate the roots and rhizomes for each sample from the ground on harvest since the plots were clearly marked and the plants were not overgrown by other vegetation in the areas that we had cleared and maintained with monthly censuses. This lack of regrowth could also indicate that the habitats were generally challenging for plant growth. We harvested roots by carefully unearthing the entire root system and shaking them to remove loose dirt in the field before thoroughly washing them in the lab.

### Traits measured

We measured traits related to salt tolerance and overall performance for each plant: height, total number of leaves, total leaf area (Li-Cor Model LI-3100 Leaf area meter: Li-Cor, Inc., Lincoln, Nebraska, USA), succulence (g water in all leaves/cm^2^ total leaf area), shoot dry biomass, root dry biomass, root to shoot ratio based on dry biomass, and total biomass at final harvest. For each plant, we used all live leaf tissue at final harvest to calculate total leaf area and succulence. We dried plants in a forced air oven at 60 °C for at least 72 h to determine shoot, root and total dry biomass. We evaluated survival and biomass as proxies for fitness to assess the degree of adaptation. These taxa have extensive clonal growth and many individuals may not flower at all in the field, but persist and spread from year to year so biomass is an important indicator of fitness^81^.

### Data analysis

We performed all statistical analyses in the R statistical programming environment version 4.0.0^82^, following the Bayesian strategy outlined by Korner-Nievergelt et al.^83^. Specifically, the basic structure of the statistical models was the same as classic frequentist approaches using the Linear-Mixed-Model (LMM) or Generalized Linear-Mixed-Model (GLMM) framework (lme4 package^84^). Then, we simulated the posterior distribution of the parameter estimates using the Bayesian simulation package arm to evaluate the probability that comparisons are truly different^85^. This contrasts with null hypothesis testing because it allows us to evaluate the probability that the differences are true given the sample data^86^. We visually checked the residuals to assess the appropriateness of the models and performed data transformations on traits as appropriate: we did not transform succulence and final height, but we performed log 10-transformation on leaf area, and log 2 transformation on shoot, root and total biomass.

We first ran all models including the covariate of rhizome weight, but it did not explain variation in any trait, so we removed this term. In addition, we evaluated the potential importance of the few different *R. japonica* and *R.* × *bohemica* haplotypes in the full model for each trait. Although we did not genotype the plants in this study, our previous assessment of these populations found that three of our populations were characterized as a single *R. japonica* haplotype (Beach 4, Marsh 2, Roadside 2) and six of the populations consisted of only six *R.* × *bohemica* haplotypes (Beach 3, Marsh 1, 3 and 4, Roadside 3 and 4; Supplementary Table S1)^52^. We ran our models for each trait with and without including the term “Taxon” defined as *R. japonica* or *R.* × *bohemica* based on the assumption that the plants in our experiment also belong to the same taxa as previously identified in those populations.

For most traits we fitted a linear-mixed model (LMM), with origin habitat type (“ORIGIN.type”), garden habitat type (“GARDEN.type”), and their interactions as fixed effects. We included origin site (“Origin.site”), garden site (“Garden.site”), and individual rhizome weight (“rhizome”) as random intercepts. For leaf number, we fitted a generalized linear mixed model (GLMM) with a negative binomial distribution, and the same model structure for independent variables.

We did not model root to shoot ratio directly, but instead we used the ratio of the estimates of mean and variance for root biomass and shoot biomass (both log-transformed) to assess probabilities within the Bayesian framework^83^.

In the LMM and GLMM models, “ORIGIN.type” and “GARDEN.type” both included beach, marsh, and roadside. The origin site, the transplant garden site, and the individual rhizome were initially included as random terms since they simply served as replicates of habitat type and individual. To avoid overfitting, we removed random effect terms that effectively explained no variance. This was true for the rhizome term for all traits and for the “Origin.site” term for number of leaves, succulence and shoot biomass (see Supplementary Table S2 for final models).

We examined the correlation matrix for each model to determine auto correlation between terms. To test the statistical uncertainty of the parameter estimates of the fixed effects of “ORIGIN.type” and “GARDEN.type”, we used 95% credible intervals (CrI), a Bayesian analogue of confidence intervals^87^. The Bayesian CrI provides a range within which there is a high (95%) probability that the true value of the parameter lies^86^. For each response variable, we obtained the model estimates from the back-transformed effect sizes. We calculated the associated 95% CrI for the model parameters based on their posterior distributions assuming non-informative prior distributions by performing 10,000 simulations using the sim function in the R package arm^83^. We used the values corresponding to the 2.5 and 97.5 quantiles of the distribution to designate the lower- and upper-boundary of the 95% CrI.

To understand how much of the variance was explained by the random and fixed effects in our models, we used several approaches. First, we used the package r2_nakagawa: Nakagawa’s R2 for mixed models^88,89^ to determine the conditional R^2^ (the variance explained by both the fixed and random effects) and marginal R^2^ (the variance explained by the combined fixed effects). The random effect variances calculated in this package are the mean random effect variances, and appropriate for mixed models with nested random effects^90^. We also used the package rptR^91^ to further evaluate the components of variance for each of the random effects separately (i.e., “Origin site” and “Transplant site”). We used the package commonalityCoefficients^92^ to examine the amount of variance explained by the separate fixed effects of the origin habitats and transplant garden habitats (i.e., “ORIGIN.type” and “GARDEN.type”). This approach does not include information about the random effects of origin site and transplant garden site which are nested within the origin type and garden type. However, this approach is valuable for evaluating the relative contribution of each separate fixed effect. We also reran the models for each trait with the fixed term “Transplant group” to evaluate the design constraint that origin and transplant garden sites were nested within Transplant groups (Supplementary methods). However, we found that the overall R^2^ and the proportion of variance explained by our main effects of interest changed very little (Supplementary Table S3).

We tested for local adaptation with two fitness proxies: total biomass and survival. We took a two-step approach. In the first step, total biomass was fitted with the LMM specified above, while survival was fitted with logistic regression. In the second step, the posterior distributions for each origin x garden habitat combination were compared to generate posterior distribution of mean and credible intervals for differences between each “local” vs. “foreign” population (sensu Korner-Nievergelt). Specifically, we randomly sampled 10,000 values from each posterior distribution. For total biomass, we calculated pairwise differences between each pair of “local” and “foreign” populations, and for survival, we calculated log-of-odds ratio.

We reported the mean, 95% CrI, and percentage of contrasts showing superior performance of plants grown in their home site compared to plants from each of the other habitats as magnitude of local adaptation. We evaluated survival overall (Japanese knotweed *s.l.*) as well as separately within *R. japonica* and *R.* × *bohemica.*

## Results

### Phenotypic response to reciprocal transplants

We found AIC values were lower for the model that did not include the taxon term for succulence and leaf area, and AIC values were very similar between models with and without the term “Taxon” for leaf number, shoot and root biomass (Supplementary Table S4). In the model for final height, including the term “Taxon” as a fixed term led to zero variance partitioned to the random effect term “Origin.site”, and the effect size estimate of taxon was similar to the estimate for origin.site in the original model that did not include “Taxon”. This likely reflected considerable co-linearity between the two terms and using “Taxon” instead of “Origin.site” only modestly improved AIC scores (3438.5 instead of 3446.0). Therefore, we found that including “Taxon” did not lead to marked improvement in the model fit. The one exception was our analysis of survival since logistic regression of “Taxon” on survival found that taxon alone explained a small amount of the variation in survival which could be important for the spread of this species complex (see below). The study was not designed to test for the effect of taxa and has limited power to do so, hence we did not include Taxon in our final models for traits and biomass. Instead, we considered the response of the complex of Japanese knotweed sensu lato as in our previous work^51^ and several other studies of Japanese knotweed^48,49,57,75^.

**Figure 3:**
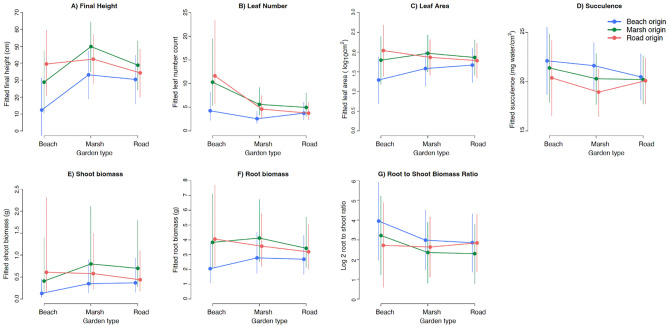
Reaction norms of (means ± 95% CrI) across three transplant habitat gardens for plants from the three habitat origins: (**A**) final height, (**B**) total number of leaves, (**C**) total leaf area of all leaves at final harvest, (**D**) succulence as measured on all leaves at final harvest, (**E**) dry shoot biomass, (**F**) dry root biomass and (**G**) dry root biomass:dry shoot biomass ratio at final harvest. Beach origin plants are depicted with blue lines, marsh origin with green and roadside origin with red.

**Figure 4:**
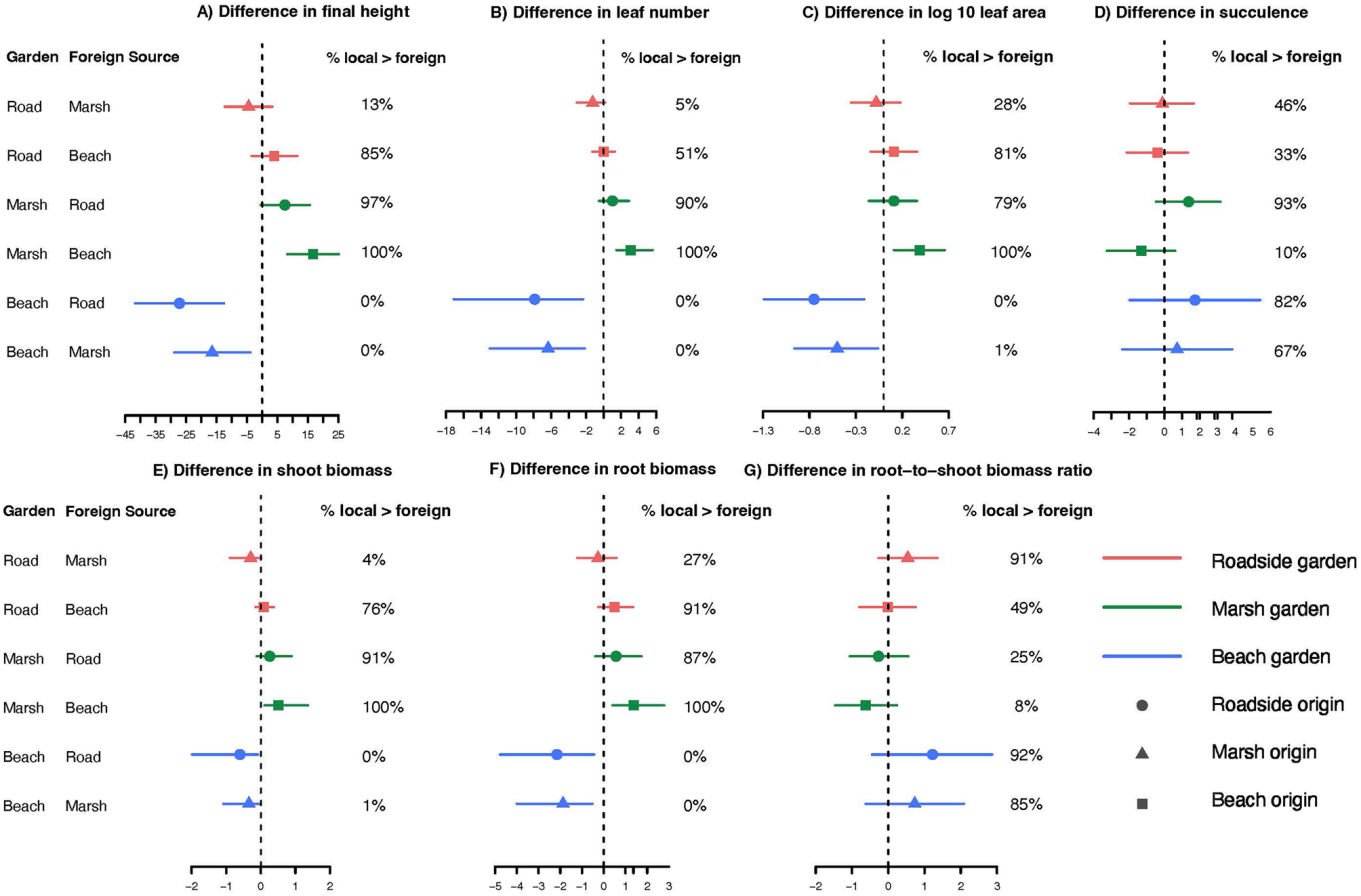
Differences in trait responses across three transplant habitat gardens for plants from the three habitat origins: (**A**) final height, (**B**) total number of leaves, (**C**) total leaf area of all leaves at final harvest, (**D**) succulence as measured on all leaves at final harvest, (**E**) dry shoot biomass, (**F**) dry root biomass and (**G**) dry root biomass:dry shoot biomass ratio at final harvest. Beach sites are depicted with blue lines, marsh sites with green and roadside sites with red.

We found that analyses of all traits resulted in large credible intervals (CrI) around the estimates of the means within origin-by-garden combinations (Fig. 3). This is probably due to the large variation in physical characteristics among transplant garden sites (Table 2). Despite this large variance, we found differences in responses depended on the origin habitat and the garden habitat. For every trait, at least one comparison showed greater than 90% probability that the differences between the compared groups were true (5 of 7 showed differences in at least one comparison with nearly 100% probability; Fig. 4).

**Table 2:** Tests for components of variance for each trait with random effects of origin site and transplant garden site, and fixed effects of origin habitat type and transplant garden habitat type.

	r2_nakagawa		rptR			commonalityCoefficients			
	Conditional r2 (random and fixed)	Marginal r2 (fixed)	Origin site (random)	Garden site (random)	Fixed effects	Unique to Origin type (fixed)	Unique to Garden type (fixed)	Common to Origin & Garden	Total
Final height	0.448	0.12	0.021 [0, 0.073]	0.421 [0.116, 0.612]	0.077 [0.035, 0.35]	0.127	0.063	0	0.186
Total leaf number	0.375	0.16	NA	0.143 [0, 0.212]	0.08 [0.039, 0.236]	0.048	0.072	0.008	0.128
Total leaf area	0.29	0.04	0.006 [0, 0.068]	0.174 [0.011, 0.335]	0.069 [0.03, 0.234]	0.066	0.075	0.006	0.147
Succulence	0.185	0.03	0.019 [0, 0.095]	0.204 [0, 0.379]	0.018 [0.01, 0.166]	0.021	0.008	0.0003	0.166
Shoot biomass	0.341	0.06	NA	0.367 [0.093, 0.561]	0.045 [0.025, 0.258]	0.093	0.023	0.002	0.119
Root biomass	0.366	0.05	0.016 [0, 0.077]	0.423 [0.117, 0.620]	0.027 [0.018, 0.299]	0.088	0.062	0.007	0.157
Total biomass	0.379	0.06	0.013 [0, 0.07]	0.427 [0.098, 0.606]	0.034 [0.022, 0.283]	0.099	0.051	0.007	0.157

The three tests of variance provide information about R^2^ of the full model versus just fixed effects (r2_nakagawa), R^2^ of the two random effects and combined fixed effects (rptR) and the contribution of each fixed effect without accounting for random effects (commonalityCoefficients). See “Methods” for more details.

In the beach gardens, plants originally from this habitat had only one-third the height, less than half the number of leaves, 30% less leaf area, one-fourth the shoot biomass and half as much root biomass as plants from the roadside habitats (Fig. 3; Table S4). Plants from the beach habitat also had half as many leaves and nearly half the root biomass of plants from the marsh habitat when grown in the beach gardens. In the marsh gardens, plants from marsh habitats had two and half times the height and twice the number of leaves as plants from beach habitats. In addition, plants from beach habitats had greater succulence than plants from the roadside habitats (but not greater than plants from marsh habitats) when grown in the marsh gardens. In roadside gardens, we found no differences among the groups for any of these traits. We also discovered that the responses of plants from marsh and roadside habitats were largely indistinguishable in any garden (Figs. 3 and 4).

These findings were supported by examining simulated values of the differences between the groups of plants for each trait (Fig. 4). In the beach habitat, beach plants were almost always shorter, had fewer leaves, had less leaf area, less root and shoot biomass than plants from marsh or roadside habitats (beach < marsh plants in 99 or 100% of the simulations). In the marsh garden, marsh plants were taller, had more leaves, greater leaf area, shoot and root biomass than beach plants in 100% of the simulations. Compared to roadside plants transplanted in the marsh garden, marsh plants were also usually taller (97% of the simulations), had more leaves (90%), greater leaf area (79%), shoot (91%) and root (87%) biomass but the differences in response were not as strong as comparisons to beach plants. In the roadside garden, again simulations did not support any differences in pairwise comparisons of plants from different habitats.

### Explanatory power of the models of phenotypic variance

Our linear mixed models explained 18 to 45% of the variation in the traits we measured (Table 2). However, we found that the majority of the variance was explained by the random effects (r2_nakagawa in Table 2) and in particular the “Garden site” which alone explained 14–43% of the variance in these traits. Only 2–8% of the variance was explained by the fixed effects of “Origin type” or “Garden type” (rptR in Table 2) which were our main interest to test the general effects of habitats. Together the fixed effects were best able to explain variance in height and number of leaves (8%) and least predictive of succulence (2%). We used the commonalityCoefficients program to further examine the amount explained by each of the fixed effects. The origin type explained twice as much of the variance as transplant garden type for height, succulence and total biomass, and three times the variance in shoot biomass, but less of the variance than transplant garden type for the number of leaves. Origin type explained a similar amount of the variance as transplant garden type for leaf area.

### The effect of transplant habitats on fitness proxies

**Figure 5:**
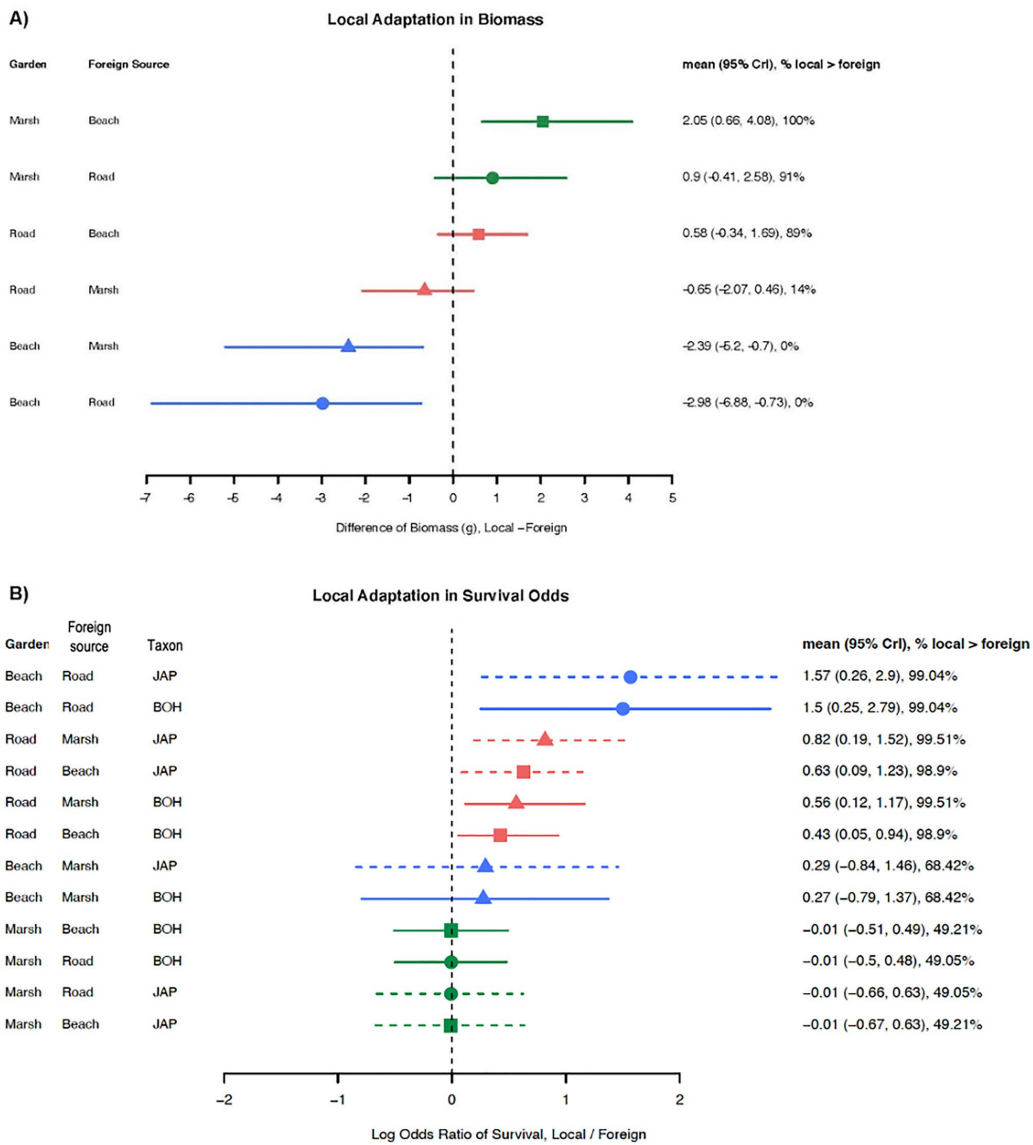
Local adaptation is supported in (**A**) marsh plants compared to beach and roadside plants grown in marsh habitats as measured by total dry biomass (g) and (**B**) survival of beach plants compared to roadside plants grown in beach habitats, and roadside plants compared to beach or marsh plants grown in roadside habitats. Survival in *R. japonica* (dashed lines) and *R.* × *bohemica* (solid lines) are indicated separately. Plants grown in beach sites are depicted with blue lines, marsh sites with green and roadside sites with red. Symbols and whiskers are differences of fitted estimates and credible intervals estimated from statistical models (see “Methods” for details).

We investigated the fitness proxies of total biomass and survival. In the beach gardens, plants from beach habitats accumulated less biomass than plants from either marsh or roadside habitats in 100% of the simulations, contrary to predictions of local adaptation. In the marsh gardens in contrast, plants from the marsh habitat accumulated more biomass than plants from beaches (100% of the simulations) and tended to grow bigger than plants from roadsides (in 90% of the simulations but the effect size was smaller; Fig. 5a). We found little support for differences in biomass among groups when grown in the roadside gardens.

Our model explained approximately 38% of the variance in total biomass. This variance was largely (i.e., 43%) determined by the random term “transplant garden site” (Table 2). The fixed effect of origin habitat type explained almost twice as much as that of transplant garden habitat type, but combined they explained only 6% of the variance in biomass (Table 2).

The model that included the main effect of “Taxon” on survival indicated that taxon alone explained a small amount of the variation in survival. However, the CrI estimate in survival odds remained nearly identical between the taxa when they were analyzed separately (Fig. 5b), and the reaction norm indicated that the two taxa simply had a different intercept. We suspect that the effect of Taxon is again due to the imbalance in our study design.

Mortality was high across the experiment, but particularly in the beach habitat garden sites (average 89% mortality; Table 3), where one site was completely washed away in a storm (PJB or Beach 2) and two other sites suffered 95–97% mortality. In both *R. japonica* and *R.* × *bohemica,* plants from marsh and roadside habitats showed an average of 2.5- to 4.9-fold decrease of survival odds when transplanted into beach habitats, while beach plants had an average 2.2–2.7-fold increase in survival odds when transplanted to either of the other habitats (Supplementary Fig. S1). Despite this high mortality overall, plants from beach habitats had higher survival in their home site than plants from roadside habitats in 99% of the simulations, but no difference in survival compared to marsh plants for both taxa (Fig. 5b). Similarly, survival of roadside plants was greater than plants from other habitats in the roadside garden and the odds were slightly higher for *R. japonica* (odds ratios 0.63 and 0.82) than *R.* × *bohemica* (odds ratios 0.43 and 0.56) plants (Fig. 5b). These findings support the predictions of local adaptation.

**Table 3:** Breakdown of mortality by group within transplant garden and average for each habitat type.

	Garden	Mortality (%)
Beaches		
1	MSH	95
2	PJB	100
3	RPB	97
4	HP	65
Average		89
Marshes		
1	CBH	29
2	CMM	94
3	WH	65
4	RHBH	57
Average		61
Roadsides		
1	ST	72
2	CMR	63
3	HL	74
4	RHC	34
Average		61

Overall, we found some support for local adaptation: plants from the marsh habitat grew bigger than plants from foreign habitats (Fig. 5a), plants from roadside habitats showed better survival when compared to foreign plants (Fig. 5b), and plants from beach habitats showed better survival compared to foreign roadside plants (Fig. 5b). Survival comparisons among plants grown in the marsh gardens did not support local adaptation.

## Discussion

In this study, we investigated how one of the world’s most invasive plants may be adapting to three different habitats on Long Island, NY. Many studies have demonstrated that differences in habitat characteristics can result in adaptive differentiation within species, even under high levels of gene flow between habitats^67,93,94^ (but see^95^). Introduced species in particular have been highlighted because they can evolve rapidly in response to novel conditions^20,96,97^. We took advantage of replicate populations to test the generality of adaptation to different habitats. Although we found wide variation among sites within habitat types, we revealed differentiation between plants from beach, marsh and roadside populations for most of these purportedly adaptive traits, as well as for measures of fitness. In addition, we found some support for local adaptation.

### Phenotypic plasticity in Japanese knotweed

Overall, the variation in phenotypes in our study was most often best explained by the local conditions of the specific transplant garden site. These findings indicate the importance of plasticity, which has often been highlighted in invasion ecology^21,33,34,98^. In our previous greenhouse study, clonal replicates of plants from both roadside and marsh sites showed significant differences in most traits and trait plasticities. The importance of transplant garden site indicates that there were important differences among our replicates of habitat type that we were able to account for in our models and allowed us to test for habitat “type” (i.e., beach, marsh or roadside more generally).

### Habitat differences

In order for adaptive differentiation to occur, habitats must select for different trait means, plasticities or relationships among traits. The beach habitat was typically open with plenty of sun. In contrast, the marsh habitats were typically under a canopy of tall (2.5–3 m), well established knotweed or *Phragmites* plants, and the roadside sites were typically under a canopy of trees. Our pairwise comparisons of plants from different habitats across transplant gardens indicated that plants were significantly different for every trait except R:S. Most of the differences were manifest in the beach common gardens where beach plants were shorter, with fewer leaves, and less biomass than plants from marsh and roadside sites. This finding of more differences in the beach habitat is similar to our previous study where we found differences in succulence and root to shoot biomass ratio only under salt addition, but not under pure water conditions^51^ (see similar results in *Borrichia frutescens*^56^). Similarly, a recent reciprocal transplant study in the salt marsh cordgrass *Spartina alterniflora* identified more extreme differences in one habitat for survival, maximum height, root to shoot biomass ratio and total biomass^67^. In these examples, the conditions that were more challenging for the plants also elicited more differences between plants.

We did find a few differences elicited in the marsh transplant gardens where marsh plants tended to be larger than the others. We expected that succulence could be important for invasion of the saline marsh habitat because the ability to become succulent, and dilute the toxic effect of concentrated salt ions, is essential for many species in saline environments^53^. For example, *Salsola kali* originating from different habitats was found to have dramatic intraspecific variation in succulence and the salt tolerant subspecies *S. kali traga* was able to increase succulence more than the non-salt tolerant *S. kali ruthenica*^99^. However, we did not find consistent responses in succulence in our previous work with these knotweed taxa. Instead, we found a lot of variation among knotweed rhizomes that gave rise to differences in succulence in response to salt treatments, including replicates from rhizomes that seemed to display no change in succulence^51^. In this study, the only difference in succulence we found was that the beach plants were more succulent than the roadside plants in the marsh transplant garden, which could indicate adaptive change in response to the beach habitat. Succulence could aid in the adaptation to water limited habitats in *Reynoutria* but could be specific to certain rhizomes or conditions that we did not explore with our current design. In addition to the possible importance of succulence, individuals may also vary in other traits that can contribute to salt tolerance through e.g. exclusion or excretion of salts or changes in water use efficiency or nitrogen use efficiency. Further studies will be required to examine the potential mechanisms of salt tolerance in these species.

The differences in phenotype elicited by these habitats could result in adaptive differentiation if there is heritable variation for these traits within the populations. We did not detect any rhizome—level variation within populations in this study. However, our power to detect this level of variation was limited by the high mortality. The random effect of origin site did not explain much of the variation in traits; however, the origin habitat type was a better predictor of variation than transplant garden habitat type for most traits.

### Signature of local adaptation

We compared the performance of local plants with that of the foreign plants for fitness proxies (total biomass and survival) in each of the common gardens to assess local adaptation. Previously, a similar reciprocal transplant study in knotweed found little support for local adaptation along a latitudinal gradient in three populations from similar temperate deciduous forest habitats^100^. Comparing responses to different habitat types on a more local scale, we found support for local adaptation in plants from the marsh: they out-performed plants from both beach and roadside habitats in biomass. This is surprising given our previous work in the greenhouse which did not support adaptive response to salt treatments. In the current study, the salt marsh plants were the only plants that accumulated more biomass in their home site. This growth advantage, however, did not translate into a survival advantage of marsh plants over foreign plants in the marsh garden. Instead, there were no differences in survival due to habitat of origin in the marsh transplant garden.

At the same time, plants from beach and roadside habitats failed to accumulate more total biomass in their home environment compared to foreign plants. Beach plants tended to allocate more resources to roots, which may have contributed to their odds of increased survival compared to foreign roadside plants. However, beach plants did not survive better than marsh plants, at least during the time frame of our study. It is unclear whether preferential allocation to roots could eventually lead to an advantage, or if this response is constrained by other factors, and the advantage could become clearer in a longer term study. In fact, plants from the beach habitat had a greater probability to grow larger in either of the away habitats. Growing on the beach on average reduced the biomass accumulation by 1.03–1.1 g, which may indicate that beach habitats are suboptimal for plant growth. On the other hand, slow biomass accumulation could be a component of an adaptive strategy on the beach. Beach plants had fewer leaves and less leaf area which may be adaptive under the extreme heat and irradiation experienced on the beaches. In that case, biomass may not be a good fitness estimate in this habitat, particularly during the first few years of establishment.

We also found support for local adaptation among roadside plants, which were better able to survive in their home sites than plants from either of the other habitats. In addition, beach plants had better survival odds when compared to roadside foreign plants grown in beach habitats. Both findings are somewhat surprising considering that plants from roadside habitats are largely indistinguishable from plants from marsh habitats for most traits that we measured.

### Sources of phenotypic differentiation

We were unable to genotype the specific plants used in this study, but in our previous work, we used cytology and AFLP markers to show that many of these populations consist of a few *R.* × *bohemica* hybrids^52^. Several studies have demonstrated that hybridization can result in significant changes in trait expression (e.g., transgressive traits) with important ecological consequences^12,16,55,101,102^. In fact, novel traits resulting from hybridization are considered an important feature that allows expansion into novel habitats in several species^103^, including in highly saline environments^55,101,104^. For example, hybrids between the invasive *Carpobrotus edulis* and the native *C. chilensis* have higher biomass in response to low salinity treatments under low nutrient conditions^105^, indicating that they may have an advantage in nutrient poor soils. These and other studies suggest that recombination of different traits may allow for rapid adaptation to new environments^103,106–108^.

Hybridization between *R. japonica* and *R. sachalinensis* to form *R.* × *bohemica* has also been suggested as an important mechanism in the Japanese knotweed *s.l.* invasion^44,76,109,110^. We found little support for differences among *R. japonica* and *R.* × *bohemica*. However, taxon explained a small amount of the variation in survival. For the long-term dynamics of the knotweed invasion, it may be meaningful that *R. japonica* plants had slightly higher survival odds than *R.* × *bohemica* hybrids in some of our field transplants. However, our study was limited to detect these differences among taxa.

Even considering that the few genotypes that have invaded these habitats may have benefited from transgressive traits, the dramatically varied response to the transplant habitats from what should only be a few genotypes is surprising. For example, the plants from Marsh 4 and Roadside 4 were identical across AFLP markers^52^, and they have almost identical survival at the roadside site. However, at the marsh transplant sites, the marsh plants had a significantly higher survival rate. Under different circumstances, this could reflect the importance of maternal effects or provisioning. However, in our study we took care to start plants with similar initial rhizome weights. In combination, these transplant gardens show that complex ecologically relevant environments elicit differences in phenotype that are not detectable by manipulation of salinity alone under controlled conditions further underscoring the importance of conducting studies in the field^58,111–113^. It could also be that we did not detect causal polymorphisms which are unlikely to be revealed with the AFLP approach. More powerful genomics approaches will be required to identify such genomic changes.

A potentially important possibility is that persistent epigenetic effects may have resulted from the hybridization process or may have been induced by exposure to these dramatically different environments^114^. We have reported higher levels of epigenetic variation in these populations compared to levels of sequence-based variation found with AFLP^52^. Epigenetic effects have been suggested as a source of phenotypic variation in ecologically relevant traits, but they have not yet been explored extensively in studies of invasive species^40,115^. In some cases, environmentally-induced epigenetic changes may be inherited by future generations^116–120^ and therefore could contribute to explaining short-term adaptation to novel environments. Moreover, epigenetic processes are an important component of hybridization events^17,40,121^.

## Conclusion

Although we found mixed support for local adaptation, this is not so unusual^100^. Leimu and Fischer^95^ performed a meta-analysis of local adaptation studies and found that plants from “home” populations outperformed the “foreign” plants in both habitat types in only 51% of the studies surveyed. These findings were independent of plant longevity, mating system, clonality or habitat type and the authors concluded that local adaptation may not be as common as it is assumed. Considering the potentially random sampling of genotypes during the invasion process, genetic drift may play a large role in shaping the evolutionary trajectory of these populations^41,42^. Further, identifying adaptive changes in a small founding population is difficult because it requires identifying the source of the invasion and comparing responses of the invaders to those of the source material^6,10,41,42^ (e.g. Refs.^122,123^).

Despite our mixed evidence, adaptive processes could still be important for most of the populations, since the responses of transplants depended on their habitat of origin. The current study confirms our findings from the greenhouse that there is phenotypic differentiation among these populations of Japanese knotweed, some of which is attributed to habitat differences. Some of the plasticity in these traits and in fitness are likely to be passive responses to resource limitation and stress, but “active” or adaptive plasticity in underlying morphological and physiological traits may help to minimize the fitness loss in these environments^122,124–126^. Whatever the mechanisms of divergence, which could include accumulation of novel mutations, drift, selection, transgressive segregation, non-genetic effects, and genetic accommodation, this study demonstrates that there is persistent phenotypic variation present in the populations of interest. This variation in ecologically important traits provides the potential for future adaptation that could increase the already high rate of spread of this species complex in North America, and in salt marsh and beach habitats in particular. Understanding the components that contribute to the success of this extensively clonal plant with little or no interindividual genetic variation will require further studies under the complex conditions of natural field environments.

### Supplementary Information

Supplementary Information.

## Data Availability

Data and code for data analysis have been submitted for review on the Dryad Digital Repository: 10.5061/dryad.wdbrv15qz.
