# Supplementary material for: Rapid phenotypic differentiation in the iconic Japanese knotweed s.l. invading novel habitats
Source: Sci Rep. 2024 Jun 25;14:14640. doi: 10.1038/s41598-024-64109-1 (PMC11199593; doi:10.1038/s41598-024-64109-1)
Supplement: Supplementary file 1 — Supplementary Information. [file 41598_2024_64109_MOESM1_ESM.pdf]

Supplementary material

Rapid phenotypic differentiation in the iconic Japanese knotweed *s.l.* invading novel habitats

Wei Yuan[1], Massimo Pigliucci[2] & Christina L. Richards[3,4*]

1. Department of Molecular Biology, Max Planck Institute for Biology, Tübingen, Germany
2. Department of Philosophy, City College of New York, NY, USA
3. Department of Integrative Biology, University of South Florida, Tampa, FL, USA
4. Plant Evolutionary Ecology group, University of Tübingen, Tübingen, Germany

*Author for correspondence

Supplementary Table S1: Site locations, taxa, and AFLP haplotypes (Richards et al., 2012).

Site	Location	Latitude	Longitude	Species	AFLP Haplotypes
**Beach**					
HP	Hunt's Point, Southold, NY	41 05.17	72 26.68	*F. japonica*	F
MSH	Mount Sinai Harbor, Port Jefferson, NY	40 57.74	73 02.58	*F. japonica/* *F. × bohemica*	F / C, D, E, G
PJB	Port Jefferson Beach, Port Jefferson, NY	40 57.88	73 03.17	*F. japonica/* *F. × bohemica*	F / A, F, G
RPB	Rocky Point beach, Rocky Point, NY	40 57.96	72 57.28	*F. × bohemica*	E
**Roadside**					
CMR	Montauk Highway, Terrell River County Park, Center Moriches, NY	40 47.98	72 46.42	*F. japonica*	F
HL	Hagerman Landing Road, Rocky Point, NY	40 57.82	72 57.29	*F. × bohemica*	E, G
RHC	Chauncey Road at Highway 24, Riverhead, NY	40 54.53	72 37.47	*F. × bohemica*	G
ST	Route 25 in Given County Park, Smithtown, NY	40 51.473	73 12.60	*F. × bohemica*	G
**Marsh**					
CBH	Crystal Brook Hollow Road Port Jefferson, NY	40 57.19	73 02.71	*F. japonica/* *F. × bohemica*	F / D, E, G, H
CMM	Terrell River County Park, Center Moriches, NY	40 47.96	72 46.41	*F. japonica*	F
RHBH	Privately owned boathouse, Peconic Bay Riverhead, NY	40 54.24	72 37.11	*F. × bohemica*	G
WH	Wertheim National Wildlife Refuge, Brookhaven, NY	40 46.23	72 53.86	*F. × bohemica*	G

Supplementary Table S2: Final models for each of the six traits.

Trait	Transformation	Type of model	Final model
Succulence	None	LMER	Origin type + Garden type + Origin type x Garden type + (Garden site)
Leaf area	log 10	LMER	Origin type + Garden type + Origin type x Garden type + (Garden site)
Total leaf number	negative bionomial	GLMER	Origin type + Garden type + Origin type x Garden type + (Garden site)
Final height	None	LMER	Origin type + Garden type + Origin type x Garden type + (Origin site + Garden site)
Shoot biomass	log 2	LMER	Origin type + Garden type + Origin type x Garden type + (Garden site)
Root biomass	log 2	LMER	Origin type + Garden type + Origin type x Garden type + (Origin site + Garden site)

Supplementary methods: Additional analyses including “Transplant group” as a fixed effect.

Methods

Our design was constrained by the fact that origin site and transplant sites are nested within levels of “Transplant group” [80]. To examine the importance of this design constraint, we also reran the LMER and GLMER models for each trait with the fixed term “Transplant group”. To properly test for the effects in this nesting design, we should ideally fit random intercepts for the sites nested within groups, but we did not have enough replication within groups to do so. We assume that fitting the fixed effect of the “Transplant group” also controls for the non-independence of the origin site and transplant site within groups (Long, 2021). By comparing the modeling with and without the fixed term of Transplant group, we evaluated how these random terms impact the main effects of interest which are the fixed effects of the habitats of the origin and transplant gardens (i.e., “ORIGIN.type” and “GARDEN.type”).

Results

When we evaluated the “Transplant group” as a fixed effect, the overall R^2 changed very little (Table S3). On average the models changed by only 0.2%. The largest change in R^2 was in the model for succulence which decreased from 29% in the original model without the effect of transplant group (Table 2) to 26% with the effect (Table S3). On average the Transplant group effect increased the amount of variance explained by fixed effects by 17%. Using commonalityCoefficients, we found that the unique contribution of transplant group was 29-65% of the variance explained by the combined fixed effects. For several traits (e.g., height, succulence, shoot root and total biomass) the majority of the variance explained by fixed effects was due uniquely to the transplant group effect (Table S3). Adding this effect also changed the amount of variance explained by “Origin type” or “Garden type”. However, when the transplant group was included, the unique contribution of origin type still explained twice as much of the variance as that of transplant garden type for shoot biomass, but a similar amount of variance as garden type for height, leaf area, succulence and total biomass. In this model, origin type explained half as much of the variance as transplant garden for the number of leaves. Therefore, we considered that the relative importance of our effects of interest (“Origin type” or “Garden type”) were not changed by this approach to the analysis.

The model without the effect of transplant group explained approximately 38% of the variance in total biomass and was largely determined by random effects, and particularly the random term “transplant garden site” (43% of the variance attributed to random effects; Table 2). Adding “transplant group” resulted in similar overall variance explained by the model (39.4%) and the largest portion of the random effects (36%) were again explained by transplant site (Table S3). The fixed effect of origin habitat type explained almost twice as much as that of transplant garden habitat type, but combined they explained only 6% of the variance in biomass (Table 2). When transplant group is included as a fixed effect, the R^2 jumps to 24% explained by combined fixed effects (according to results of r2_nakagawa, Table S3) and the effect of origin habitat type still explains more than that of transplant garden habitat type (19% compared to 13% of the variance due to fixed effects which translates to approximately 4% and 3% of the overall variance in this model).

Supplementary Table S3. As with table 2 in the main text, we provide tests for components of variance for each trait with random effects of origin site and transplant site, and fixed effects of origin habitat type and transplant garden habitat type as well as the fixed effect of transplant group. The three test of variance provide information about R^2 of the full model versus just fixed effects (r2_nakagawa), R^2 of the two random effects and combined fixed effects (rptR) and the contribution of each fixed effect without accounting for random effects (commonalityCoefficients). See methods for more details.

	r2_nakagawa		rptR		
	conditional r2 (random and fixed)	marginal r2 (fixed)	Origin site (random) [CI]	Garden site (random) [CI]	Fixed effects [CI]
Final height (396)	0.466	0.334	0.019 [0, 0.072]	0.3 [0.032, 0.497]	0.24 [0.135, 0.563]
Total leaf number (395)	0.374	0.364	NA	0 [0, 0]	0.235 [0.184, 0.321]
Total leaf area (395)	0.264	0.231	0.009 [0, 0.06]	0.165 [0, 0.362]	0.136 [0.077, 0.365]
Succulence (372)	0.185	0.067	0.015 [0, 0.095]	0.253 [0.002, 0.074]	0.045 [0.038, 0.334]
Shoot biomass (395)	0.334	0.275	NA	0.208 [0.005, 0.414]	0.226 [0.116, 0.483]
Root biomass (395)	0.382	0.209	0.015 [0, 0.064]	0.379 [0.045, 0.588]	0.148 [0.071, 0.528]
Total biomass (395)	0.394	0.239	0.013 [0, 0.057]	0.361 [0.027, 0.056]	0.169 [0.086, 0.535]

	**commonalityCoefficients**							
	Unique to Transplant group (fixed)	Unique to Origin type (fixed)	Unique to Garden type (fixed)	Common to Group & Origin type	Common to Group & Garden type	Common to Origin type & Garden type	Common to Group, Origin & Garden type	Total
Final height	0.175	0.055	0.062	0.0713	0.0003	0.003	-0.006	0.359
Total leaf number	0.075	0.039	0.076	0.0091	-0.0046	-0.002	0.0097	0.202
Total leaf area	0.060	0.042	0.056	0.0235	0.0189	-0.002	0.0073	0.147
Succulence	0.053	0.012	0.011	0.009	-0.0038	0.002	-0.002	0.029
Shoot biomass	0.155	0.042	0.017	0.0507	0.0075	0.001	0.0013	0.119
Root biomass	0.103	0.048	0.047	0.0402	0.0156	-0.001	0.0081	0.157
Total biomass	0.121	0.052	0.036	0.0477	0.0148	-0.0004	0.0072	0.157

Supplementary Table S4: Model comparisons with and without the term “Taxon” using the Linear_Mixed-Model (LMM) or Generalized Linear-Mixed-Model (GLMM) framework.

	df	AIC	Δ_{AIC}
**Succulence (N=373)**			
Succulence ~ SOURCE.type+GARDEN.type+SOURCE.type:GARDEN.type + (1|Transplant.site)	362	2382.5	0
Succulence ~ Taxon+SOURCE.type+GARDEN.type+SOURCE.type:GARDEN.type + (1|Transplant.site)	361	2382.7	+0.2
**Leaf area (N=395)**			
Leaf area~SOURCE.type+GARDEN.type+SOURCE.type:GARDEN.type +(1|Transplant.site)	384	862.3	0
Leaf area~Taxon+SOURCE.type+GARDEN.type+SOURCE.type:GARDEN.type + (1|Transplant.site)	383	863.6	+1.3
**Total leaf number (N=395)**			
TLN ~ SOURCE.type+GARDEN.type+SOURCE.type:GARDEN.type + (1|Transplant.site)	384	2072.3	0
TLN ~ Taxon+SOURCE.type+GARDEN.type+SOURCE.type:GARDEN.type + (1|Transplant.site)	383	2071.7	-0.6
**Final height (N=396)**			
Final height ~ SOURCE.type+GARDEN.type+SOURCE.type:GARDEN.type+ +(1|Origin.site)+(1|Transplant.site)	384	3446.0	0
Final height ~Taxon+SOURCE.type+GARDEN.type+SOURCE.type:GARDEN.type +(1|Transplant.site)	384	3438.5	-7.5
**Shoot biomass (N=395)**	384	1670.1	0
Shoot biomass ~ SOURCE.type+GARDEN.type+SOURCE.type:GARDEN.type + (1|Transplant.site)			
Shoot biomass ~ Taxon+SOURCE.type+GARDEN.type+SOURCE.type:GARDEN.type + (1|Transplant.site)	383	1669.9	-0.2
**Root biomass (N=395)**	383	1081.9	0
Root biomass ~ SOURCE.type+GARDEN.type+SOURCE.type:GARDEN.type +(1|Origin.site)+(1|Transplant.site)			
Root biomass ~ Taxon+SOURCE.type+GARDEN.type+SOURCE.type:GARDEN.type +(1|Origin.site)+(1|Transplant.site)	382	1079.7	-2.2

Supplementary Figure S1. Evidence of differences in survival across habitats.

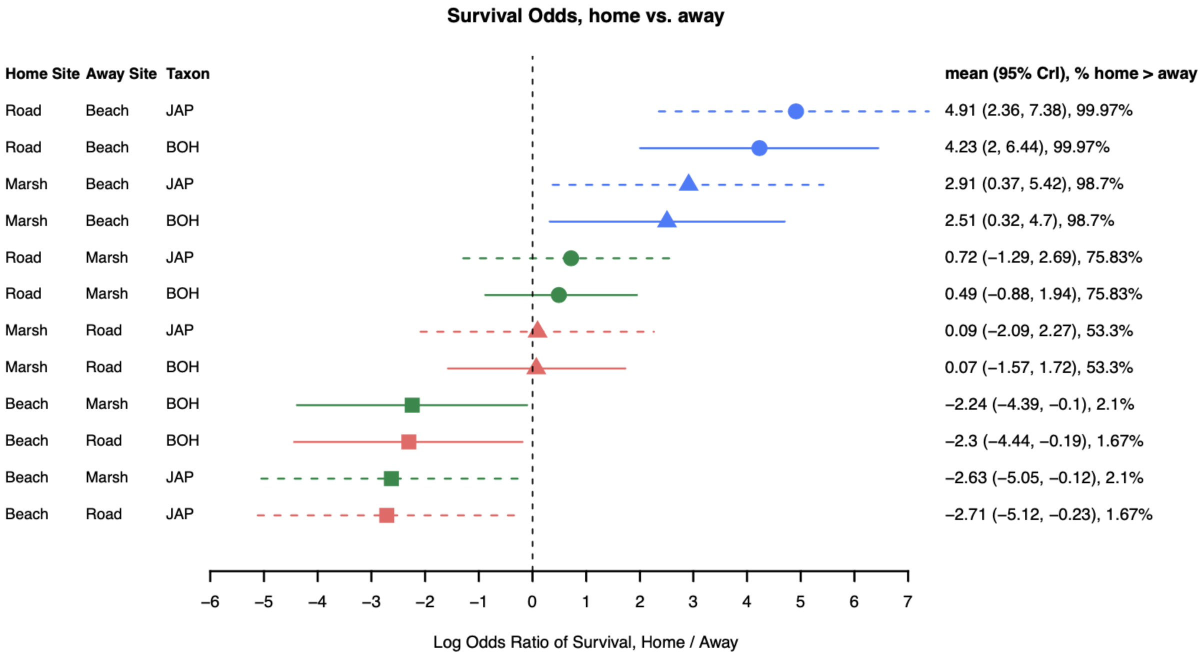

Figure S1. Evidence of differences in survival across habitats. Marsh and roadside plants from both taxa grow better in their home compared to beach habitats while beach plants grow better in marsh and roadside habitats than their home habitat. Plants grown in beach sites are depicted with blue lines, marsh sites with green and roadside sites with red. *Reynoutria japonica* is indicated with broken lines, *R.* × *bohemica* with solid lines.

Supplementary References

Richards, C. L., Schrey, A. W., and Pigliucci, M. (2012). Invasion of diverse habitats by few Japanese knotweed genotypes is correlated with epigenetic differentiation. *Ecol. Lett.* 15, 1016–1025. doi: 10.1111/j.1461-0248.2012.01824.x
